# Supplementary material for: Effects of unsaturated fatty acids (Arachidonic/Oleic Acids) on stability and structural properties of Calprotectin using molecular docking and molecular dynamics simulation approach
Source: PLoS One. 2020 Mar 26;15(3):e0230780. doi: 10.1371/journal.pone.0230780 (PMC7098580; doi:10.1371/journal.pone.0230780)
Supplement: S1 Table — (DOCX) [file pone.0230780.s001.docx]

**“S1 Table”**. MMPBSA free energies of Calprotectin-AA, Calprotectin-OA complexes and the Calprotectin alone.

| NO. | Type | van der waal (kJ/mol) | electrostatic (kJ/mol) | Polar salvation (kJ/mol) | Non-polar salvation (kJ/mol) | Binding energy (kJ/mol) |
| --- | --- | --- | --- | --- | --- | --- |
| 1 | Calprotectin –AA | -607.7 ± 6.0 | -454.3 ± 3.2 | 959.9 ± 6.6 | -72.86 ± 3.0 | -174.9 ± 5.4 |
| 2 | Calprotectin –OA | -581.9 ± 5.7 | -267.2 ± 3.7 | 835.7 ± 7.1 | -68.9 ± 3.4 | -82.4 ± 6.3 |
| 3 | Calprotectin | -659.6 ± 4.9 | -1000.9 ± 3.9 | 1477 ± 7.8 | -85.7 ± 2.9 | -269.2 ± 5.7 |
