# Supplementary material for: Effects of unsaturated fatty acids (Arachidonic/Oleic Acids) on stability and structural properties of Calprotectin using molecular docking and molecular dynamics simulation approach
Source: PLoS One. 2020 Mar 26;15(3):e0230780. doi: 10.1371/journal.pone.0230780 (PMC7098580; doi:10.1371/journal.pone.0230780)
Supplement: Attachment — Submitted filename: Response to the Reviewer1.docx [file pone.0230780.s002.docx]

**Response to the Reviewers:**

We truly appreciate the reviewer’s valuable and constructive comments. We have adopted all the suggestions in our revised manuscript.

Reviewer 1:

The manuscript "Effects of unsaturated fatty acids (Arachidonic/Oleic Acids) on stability and structural properties of Calprotectin using Molecular Docking and Molecular Dynamics simulation approach" by Alireza Farasat et al. described the effects of fatty acids on Calprotectin.
I recommend the manuscript for publication in PLOS ONE after making minor changes in the text.
1. The Authors should avoid acronyms in the abstract.

**Reply:** In the abstract part, the acronyms were omitted.

2. My main remarks concern the text in manuscript to Figure 3 on pages 9 and 10. Phe68 and Thr87 are not present in Figure 3A. Glu69 must be replaced by Gln69 in all places. ILe60 is not written correctly, change to Ile60. Phe68 and Thr87 are not common. IL72 must be replaced with Leu72.

**Reply:** The above changes were done.

3. Further comments on the text.
page 10. "As shown in the figure, the beta-sheet..." What figure?
page 10. "Previous studies proved...." Reference is absent.

**Reply:** The aforementioned changes were done.

Reviewer 2: The authors present a paper in which they study interactions of fatty acids with calprotein. The authors study an important topic and their computational approach is appropriate. However, the presented data on structural changes does not support the conclusions. Specifically, the presented values in declined beta-sheet and helix content are very similar and no error estimates for these values are given. The authors should repeat the simulations of apo calprotein and with AA/OA complexes and calculate standard errors based on the results of the two independent simulations. This is always a good practice in molecular dynamics simulations. Additionally, comparison to the results of experimental structural content in reference 20 should be done. I recommend the publication of this paper after additional simulations and statistical analysis, as well correction of the text below.

1. Correct capitalization of "van der Waals" and "electrostatic" should be used.

**Reply:** Capitalization was corrected.

2. the beta symbol should be used instead of "beta" in beta-sheets.

**Reply:** The β symbol was applied to the manuscript.

3. On page 4 sentence “Moreover, the Calprotectin has the ability
to bind UFAs comprising AA, Linoleic acid (LA) and Oleic acid (OA) in a calcium dependent
manner which is considered as the main fatty acid carrier of neutrophil with high binding affinity
potential.” is a bit weird, I propose to replace it with
“The calprotectin is considered to be the main fatty acid carrier of neutrophils with high binding affinity
potential and can bind UFAs such as AA, Linoleic acid (LA) and Oleic acid (OA) in a calcium dependent manner. “

**Reply:** On page 4, the sentence was replaced with your desired phrase.

4. Also in the next sentence on page 4, “The following attachment” should be replaced with “This binding” for clarity.

**Reply:** It was replaced with “This binding” phrase.

5. Page 6 end of introduction “(TLR4, RAGE, ....)” replace "… " with something else or remove

**Reply:** It was removed.

6. In CaCl2 2 should be subscripted, as in all chemical formulas.

**Reply:** It was done.

7. "H-bond" abbreviation should be spelled out when it is introduced and added to the list of abbreviations.

**Reply:** It was done.

8. On page 10 in sentence “As shown in the figure, the beta-sheet and helix content declined.” the figure number is missing. Also right after this sentence, "Such a way” does not fit with the text, I propose to remove it.

**Reply:** It was removed based on your valuable comment.

9. Also on page 10 the sentence "The percentage of turn structure was increased from 14% to 18% in Calprotectin-OA complex, the percentage of coils in Calprotectin was 25% while, in Calprotectin-OA complex has been reached to 26.4%" this sentence should be rewritten for clarity, please specify what systems are compared for values 14% and 18%.

**Reply:** Changes were done.

10. Reference 31 is missing journal name volume and pages.

**Reply:** As we checked the google scholar, unfortunately the aforementioned article, doesn’t have volume no., but the pages were added.

11. The resolution of the figures should be improved before publication.

**Reply:** The resolution of the figures was improved.
